# Oral microbiome correlates with selected clinical biomarkers in individuals with no significant systemic disease

**DOI:** 10.3389/fcimb.2023.1114014

**Published:** 2023-03-31

**Authors:** Mi Young Lim, Jung-Ha Kim, Young-Do Nam

**Affiliations:** ^1^ Personalized Diet Research Group, Korea Food Research Institute, Jeollabuk-do, Republic of Korea; ^2^ Department of Family Medicine, Chung-Ang University Hospital, Chung-Ang University College of Medicine, Seoul, Republic of Korea

**Keywords:** oral microbiome, clinical biomarker, metabolism, complete blood count, population study, healthy individuals

## Abstract

The oral microbiome is an important component of the microbiome in the human body. Although the association of the oral microbiome with various diseases, including periodontitis and cancer, has been reported, information on how the oral microbiome is related to health-related indicators in healthy populations is still insufficient. In this study, we examined the associations of the oral microbiome with 15 metabolic and 19 complete blood count (CBC)-based markers in 692 healthy Korean individuals. The richness of the oral microbiome was associated with four CBC markers and one metabolic marker. Compositional variation in the oral microbiome was significantly explained by four markers: fasting glucose, fasting insulin, white blood cell count, and total leukocyte count. Furthermore, we found that these biomarkers were associated with the relative abundances of numerous microbial genera, such as *Treponema*, *TG5*, and *Tannerella*. By identifying the relationship between the oral microbiome and clinical biomarkers in a healthy population, our study presents a direction for future studies on oral microbiome-based diagnosis and interventions.

## 1 Introduction

The human oral cavity harbors the second most diverse microbial community, after the intestine. The oral microbiome consists of more than 700 different species of bacteria, along with viruses, fungi, and archaea (Lamont et al., 2018). This microbial community plays various roles in maintaining not only oral health but also systemic health. Dysbiosis of the oral microbiome is associated with dental caries, periodontitis, gingivitis, and oral cancer (Huang et al., 2011; Liu et al., 2012; Jiang et al., 2018; Yang et al., 2018). Additionally, oral microbial dysbiosis is linked to inflammatory bowel disease, diabetes, rheumatoid arthritis, and atherosclerosis (Said et al., 2014; Fak et al., 2015; Long et al., 2017; Chen et al., 2018).

The oral microbiome is associated with these various diseases; therefore, the composition of the oral microbiome has the potential to yield non-invasive biomarkers for diseases. To identify oral microbiome biomarkers that are differentially abundant between healthy and disease states, it is necessary to first collect oral microbiome samples from healthy individuals and to characterize their compositions. Several studies have indeed examined healthy oral microbiomes (Zaura et al., 2009; Human Microbiome Project, 2012; Nearing et al., 2020) and reported relationships between the oral microbiome and specific factors that possibly influence oral microbiome composition, such as smoking (Wu et al., 2016), alcohol consumption (Fan et al., 2018), vegan diet (Hansen et al., 2018), and ethnicity (Mason et al., 2013). Recently, Nearing et al. found that variations in the oral microbiome of 1,049 healthy Atlantic Canadians was associated with age, sex, and waist-hip ratio; however, none of 41 different variables, including dietary factors, lifestyle, and anthropometric factors explained > 2% of the variation in the oral microbiome, suggesting that there is no strong confounding factor to be concerned with regarding biomarker detection (Nearing et al., 2020). Nonetheless, whether the oral microbiome is associated with a wide range of hematological parameters used to assess overall health conditions, even in healthy individuals, has not been examined. If there are microbial changes along with specific test measures in healthy individuals, the oral microbiome profile can be used as a biomarker for early diagnosis.

In this study, we investigated the characteristics of oral microbiome composition in a healthy Korean adult population. To determine the effect of health conditions on the cohort’s oral microbiome, we assessed whether 30 hematological parameters (11 metabolic markers and 19 complete blood count [CBC]-based markers) and 4 additional metabolic biomarkers (body mass index, waist circumference, systolic blood pressure, and diastolic blood pressure) were associated with the oral microbiome profiles.

## 2 Materials and methods

### 2.1 Study population

As part of an ongoing Korean microbiome project (Lim et al., 2021) designed to determine the composition of the gut and oral microbiomes in 10,000 Koreans who were healthy or had metabolic diseases and to identify associations of the microbiome with health and disease, we collected saliva samples from 692 apparently healthy individuals living in the Seoul metropolitan area in 2021. The exclusion criteria for the selection of apparently healthy volunteers were as follows: use of antibiotics in the last 3 months; history of major gastrointestinal surgery; any active uncontrolled gastrointestinal disorders or diseases; previous cancer diagnosis; chronic clinically significant cardiovascular, pulmonary, renal, or hepatic disease; pregnant or breastfeeding women. This study was approved by the Institutional Review Board of the Chung-Ang University Hospital (2070-005-429). All the participants provided written informed consent.

### 2.2 Clinical assessment

Participant data on 15 metabolic and 19 CBC-based markers were collected (**Supplementary Table 1**). The body weight and height of the participants were measured in light clothing without shoes using a BSM 330 (Biospace Co. Seoul, Korea). Body mass index (BMI) was calculated as weight divided by the square of height (kg/m^2^). Waist circumference was measured at the midpoint between the lowest rib and the iliac crest, while the subject was standing. Each subject was stabilized for more than 10 min, after which their blood pressure was measured using an automatic blood pressure system (FT-500R, Selvas Healthcare, Seoul, Korea) in the sitting position. Blood samples were collected after overnight fasting. Metabolic-based markers, including aspartate transaminase (AST), alanine transaminase (ALT), gamma-glutamyltransferase (GGT), creatinine, total cholesterol (TotalC), high-density lipoprotein cholesterol (HDLC), triglycerides (TG), low-density lipoprotein cholesterol (LDLC), fasting glucose, and fasting insulin, were measured using the ADVIA 1650 chemistry analyzer (Siemens, Tarrytown, NY, USA). The estimated glomerular filtration rate (eGFR) was calculated with the 2009 CKD-EPI creatinine equation. CBC-based markers were determined using the ADVIA 120 automated hematology analyzer (Siemens, Tarrytown, NY, USA). Outliers that were outside the lower and upper limits of 1.5 times the interquartile range were replaced with the 5th and 95th percentile values, respectively, to reduce the effect of outliers on the statistical analysis, especially in regression analysis.

### 2.3 Saliva sample collection, DNA extraction, 16S rRNA gene sequencing, and sequencing data analysis

Saliva samples from the participants were collected on the day of the clinical assessment. Participants were asked not to eat, drink, or brush their teeth for 1 h before saliva sampling. Participants rinsed their mouth with water and then spit 2–5 mL of unstimulated whole saliva directly into a 25-mL conical tube (Eppendorf, Hamburg, Germany). After collection, saliva samples were immediately frozen and stored at −80°C until DNA extraction.

Microbial DNA from saliva samples was isolated using the QIAamp DNA Microbiome Kit (Qiagen, Hilden, Germany), according to the manufacturer’s instructions (Qiagen, 2014). Briefly, frozen saliva samples were thawed on ice and vortexed vigorously. Next, 1 mL sample was transferred to a 2-mL tube and incubated with 500 μL buffer AHL (provided with the kit) for 30 min. After centrifugation at 10,000 *g* for 10 min, the supernatant was removed. Thereafter, samples were transferred to the QIAcube, and the subsequent steps were performed on a QIAcube (Qiagen). The total DNA was eluted in 50 μL AVE buffer and stored at −20 °C until use.

Library preparation of the V3–V4 region of the 16S rRNA gene was performed following the 16S Metagenomic Sequencing Library Preparation Illumina protocol (Part # 15044223 Rev. B, Illumina, San Diego, CA, USA). Libraries for each sample were sequenced using the Illumina MiSeq platform (Illumina). The amplicon reads with five or more mismatches to the primer sequence were discarded, and the primer parts were removed from the remaining reads. Using the DADA2 pipeline (Callahan et al., 2016) of QIIME2 (Bolyen et al., 2019), sequence quality control and feature table construction were performed *via* the “qiime dada2 denoise-paired” command with default setting except for “–p-trunc-len-f 270” and “–p-trunc-len-r 220”. Taxonomy was assigned using a naive Bayesian classifier (Bokulich et al., 2018) trained against the V3–V4 fragments of the Greengenes 13_8 99% operational taxonomic unit dataset (Desantis et al., 2006) *via* “qiime feature-classifier classify-sklearn” command.

### 2.4 Statistical analyses

Alpha diversity measures, including observed features (richness) and Shannon (diversity) index at the genus and amplicon sequence variant (ASV) levels, were calculated using the microbiome package in R (Lahti and Shetty, 2017). Associations between alpha diversity measures and clinical biomarkers were analyzed using linear regression, adjusting for age and sex. Using the “adonis2” function in the “vegan” R package, permutation multivariate analysis of variance (PERMANOVA) based on genus-level Aitchison distance was performed to estimate the variation explained by each biomarker, while controlling for age and sex (Oksanen et al., 2013). The associations between microbial taxon (from phylum to genus) and each biomarker were calculated using linear regression, with age and sex included as covariates. The regression analysis was performed on the log-2 transformed relative abundance of the taxa present in at least 10% of samples and the standardized levels of the biomarkers. In this analysis, corrections for multiple testing were performed for each taxonomic rank. Clustering of the oral microbiome was performed using Dirichlet multinomial mixtures on the rarefied genus level count data in the R package “DirichletMultinomial” (Morgan, 2022). The optimal number of clusters was selected based on the Bayesian information criterion. Principal coordinate analysis based on Bray–Curtis distance was performed to visualize the two clusters. Differentially abundant genera between clusters were identified using linear discriminant analysis effect size (LEfSe) analysis (Segata et al., 2011). Biomarkers associated with the clusters were identified using the Wilcoxon test. All *P*-values were adjusted for multiple comparisons using the Benjamini–Hochberg method. The results were considered significant at a false discovery rate (FDR) < 0.05, unless otherwise stated.

## 3 Results

### 3.1 Composition of the oral microbiome

**Figure 1:**
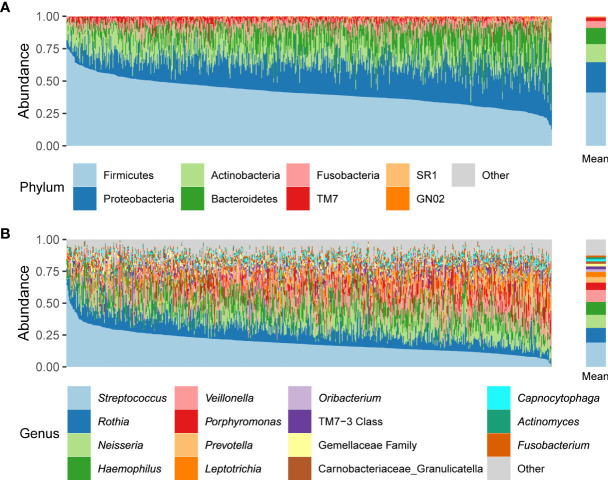
Relative abundances of phyla **(A)** and genera **(B)** across all participant samples. The left panels indicate the oral microbiome composition of each individual; the right panels indicate the mean relative abundances.

We characterized the oral microbiome composition in 692 Korean adults. At the phylum level, Firmicutes, Proteobacteria, Actinobacteria, Bacteroidetes, and Fusobacteria were the five most abundant taxa (41.2%, 23.3%, 14.1%, 12.4%, and 5.3% of the average relative abundance, respectively) (**Figure 1A**). At the genus level, the five most abundant genera across all samples were *Streptococcus, Rothia, Neisseria*, *Haemophilus*, and *Veillonella* (19.1%, 11.4%, 10.3%, 10.1%, and 9.4% of the average relative abundance, respectively) (**Figure 1B**).

We also characterized the core microbiome that appeared in at least 95% of individuals at the genus and ASV levels, because the core microbiome may play essential roles in human oral health, and thus can be a target for bacterial isolation and its functional research. We identified 28 core genera, including the five most abundant genera mentioned above (**Supplementary Table 2**). At the ASV level, 10 ASVs—one *Peptostreptococcus* ASV, one *Campylobacter* ASV, one *Haemophilus* ASV, one *Oribacterium* ASV, one *Gemellaceae* family ASV, one *Granulicatella* (*Carnobacteriaceae* family) ASV, four *Streptococcus* ASVs—were found in more than 95% of individuals (**Supplementary Table 2**).

### 3.2 Associations between microbial diversity and biomarkers

Next, we calculated the alpha diversity measures, including the observed features and Shannon index at the genus and ASV levels. In this population, the richness values measured as observed features were 60.7 ± 10.6 at the genus level and 236.8 ± 69.2 at the ASV level. The Shannon index values were 2.6 ± 0.3 at the genus level and 3.86 ± 0.4 at the ASV level.

The metabolic and CBC biomarkers that were significantly associated with alpha diversity measures were identified using linear regression analysis, adjusting for age and sex. One metabolic biomarker (fasting glucose) and three CBC biomarkers (white blood cell count [WBC], total leukocyte count [TLC], and absolute neutrophil count [ANC]) were positively associated with the number of observed genera (FDR < 0.05). One CBC biomarker (red blood cell distribution width, RDW) was negatively associated with the number of observed genera (FDR < 0.05) (**Table 1**). Similarly, we observed positive associations of the number of observed ASVs with WBC, TLC, and fasting glucose, as well as a negative association with RDW (FDR < 0.05) (**Table 1**). Unlike richness, the Shannon diversity index showed no significant association with metabolic and CBC biomarkers.

**Table 1:** Significant associations of the number of observed features (at the genus and ASV levels) with different biomarkers.

Alpha diversity	Variable	Biomarker type	Estimate	Standard error	Statistic	*P*-value	False discovery rate
Observed genera	WBC	CBC	0.9856	0.2700	3.6497	0.0003	0.0096
	Fasting glucose	Metabolic	0.0926	0.0281	3.2892	0.0011	0.0157
	RDW	CBC	-1.8953	0.5902	-3.2111	0.0014	0.0157
	TLC	CBC	0.0021	0.0007	3.0424	0.0024	0.0192
	ANC	CBC	0.0011	0.0004	2.9969	0.0028	0.0192
Observed ASVs	WBC	CBC	6.1393	1.7748	3.4591	0.0006	0.0101
	RDW	CBC	-13.0538	3.8725	-3.3709	0.0008	0.0101
	TLC	CBC	0.0151	0.0045	3.3379	0.0009	0.0101
	Fasting glucose	Metabolic	0.5365	0.1851	2.8982	0.0039	0.0329

ANC, absolute neutrophil count; ASV, amplicon sequence variant; CBC, complete blood count biomarker; Hb, hemoglobin; RDW, red blood cell distribution width; TG, triglycerides; WBC, white blood cell count; TLC, total leukocyte count.

**Figure 2:**
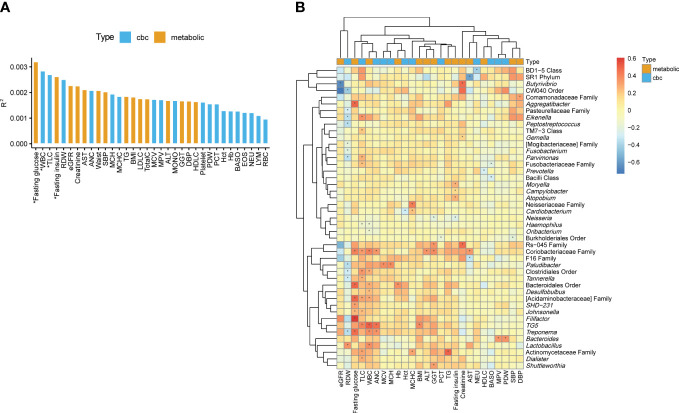
Associations of the oral microbiome with different biomarkers. **(A)** Effect size of biomarkers on the oral microbiome composition based on Aitchison distance after adjusting for age and sex. Bars are colored by the type of biomarkers. *, false discovery rate (FDR) < 0.1. **(B)** Heatmap of the association between the biomarkers and the relative abundances of genera. The color of the heatmap shows the regression coefficient estimates adjusted for age and sex. *, FDR < 0.05.

To determine the biomarkers significantly associated with variation of the oral microbiome at the genus level, we performed PERMANOVA and quantified the extent of microbiome composition variance explained by each biomarker, adjusted for age and sex, where FDR < 0.1 was considered significant. Fasting glucose (R^2 =^ 0.0032, FDR=0.068) and fasting insulin levels (R^2 =^ 0.0026, FDR=0.085) among the metabolic biomarkers and WBC count (R^2 =^ 0.0028, FDR=0.085) and TLC (R^2 =^ 0.0027, FDR=0.085) among the CBC biomarkers significantly contributed to variance in the oral microbiome compositions (**Figure 2A**).

### 3.3 Associations between biomarkers and individual oral microbial abundances

We determined the associations of metabolic and CBC biomarkers with microbial features at different taxon levels (from phylum to genus) after correcting for age and sex. We observed 249 significant associations between biomarkers and microbial taxa (phylum level: 35; class: 40; order: 38; family: 56; genus: 80) (FDR < 0.05). Focusing on the genus-level associations (**Supplementary Table 3**), an unclassified genus in the family *Coriobacteriaceae* showed the largest number of associations with biomarkers (four metabolic and three CBC biomarkers; **Figure 2B**). The genera *TG5*, *Treponema*, and *Paludibacter* showed associations with more than three biomarkers. In addition, unclassified genera in the families *Actinomycetaceae* and *Acidaminobacteraceae* and in the orders Bacteroidales and Clostridiales were significantly associated with at least three biomarkers (**Figure 2B**).

Herein, TLC was associated with the largest number of genera, including positive associations with 12 genera (e.g., *TG5*, *Parvimonas*, *Dialister*, and *Eikenella*) and a negative association with one genus (*Haemophilus*). Similarly, WBC count was associated with 10 genera, of which 8 were positively associated. RDW were associated with 12 genera. However, unlike the two above-mentioned biomarkers (TLC and WBC), RDW was negatively associated with 11 genera (e.g., *Treponema*, *Eikenella*, and *Tannerella*), whereas it was positively associated with only one genera (*Lactobacillus*). Among metabolic biomarkers, fasting glucose showed only positive associations with eight genera, and the pattern of associations was similar to that of TLC and WBC count. Fasting insulin showed a negative association with *Neisseria* and positive associations with *Moryella*, *Campylobacter*, and *Atopobium* (**Figure 2B**).

### 3.4 Clusters of oral microbiome and their association with biomarkers

**Figure 3:**
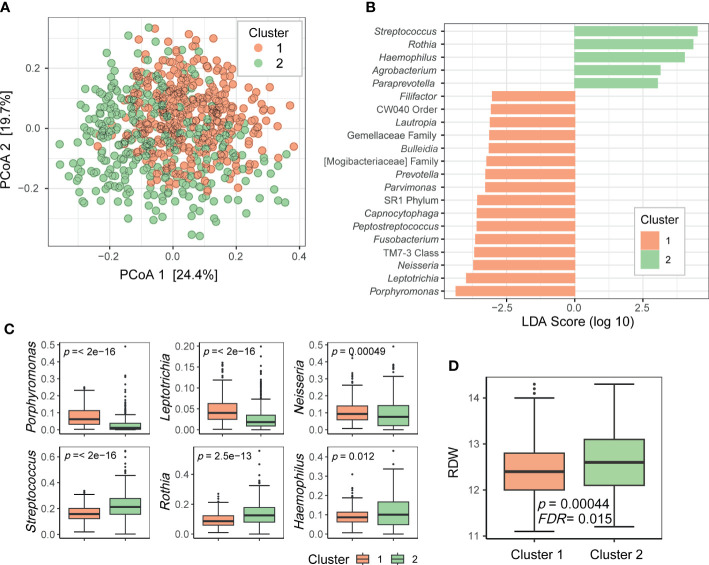
Clusters of the oral microbiome. **(A)** Principal coordinate analysis plot of the genus-level data based on the Bray–Curtis distance. Dots are colored by cluster. **(B)** Differentially prevalent genera in two clusters based on linear discriminant analysis effect size (LEfSe) analysis. LDA scores (log 10) > 3 are shown. **(C)** Boxplots of selected genera with differential relative abundance between clusters from LEfSe analysis. *P*-values on each plot were calculated using the Wilcoxon rank-sum test. **(D)** Red blood cell distribution width (RDW) levels associated with oral microbiome clusters. *P*-value was calculated using the Wilcoxon rank-sum test. FDR value was estimated to control for multiple testing.

To determine whether the oral microbiome could be stratified into several groups, such as enterotypes of the gut microbiome, we clustered the relative abundance data of the genera present in the oral microbiome. Using the Dirichlet multinomial mixture modeling method, the oral microbiome samples were divided into two clusters (1 and 2; **Figure 3A**). LEfSe analysis revealed that cluster 1 was enriched with 16 taxa including *Porphyromonas*, *Leptotrichia*, and *Neisseria*, whereas cluster 2 was enriched with 5 taxa including *Streptococcus*, *Rothia*, and *Haemophilus* (**Figure 3B**). The top three most differentially abundant genera in each cluster are shown in **Figure 3C**. Subsequently, we searched for the biomarkers associated with these oral microbiome clusters. Among the health-related biomarkers and age, only RDW levels differed between the two clusters (two-sided Wilcoxon test, FDR = 0.015; **Figure 3D**).

## 4 Discussion

We examined the characteristics of the oral microbiome in 692 healthy Korean individuals. The variation in each individual’s oral microbiome composition was prominent (**Figure 1**), but 28 genera and 10 ASVs were commonly found in at least 95% of individuals (**Supplementary Table 2**). The core genera covered 95% of the mean relative abundance. Among the core genera, the sum of the mean relative abundances of *Streptococcus*, *Rothia*, *Neisseria*, *Haemophilus*, and *Veillonella* accounted for 60.3% of the total, whereas 12 core genera had < 1% mean relative abundance. This result indicated that the genera shared among the individuals occupied the majority of the oral microbiome composition, but the mean relative abundance of each core genus was diverse. At the ASV level, the number of core ASVs was only 10, and the sum of their mean relative abundances was only 23.2% (**Supplementary Table 2**), which indicated high variability of ASV composition between individuals.

This study focused on the associations between the oral microbiome and health-related biomarkers to determine whether the oral microbiome influence a particular health biomarker, even in healthy individuals. Age and sex were included as covariates in the analysis to avoid potential biases. Among the CBC biomarkers, WBC and TLC showed significant associations with alpha and beta diversities of the oral microbiome (**Table 1** and **Figure 2A**). In addition, they were associated with a large number of genera (**Figure 2B**). WBC count and TLC are important indicators of the state of the immune system, especially in infections, inflammation, autoimmune diseases, and immune deficiencies. The oral microbiome of patients with immune-related diseases differs from that of healthy individuals. For example, in patients with inflammatory bowel disease, the diversity of the oral microbiome was lower than that of healthy controls, and the abundances of the phyla Fusobacteria and Firmicutes were lower than those in healthy controls (Docktor et al., 2012). In patients with rheumatoid arthritis, the prevalence of anaerobes, such as *Lactobacillus salivarius*, *Atopobium* spp., and *Cryptobacterium curtum*, and the reduction of aerobes, such as *Neisseria* spp., was observed in comparison to that in healthy controls (Zhang et al., 2015). RDW was another CBC biomarker significantly associated with the oral microbiome in terms of richness, relative abundance of genera, and clusters based on microbiome composition (**Table 1**, **Figures 2B** and **3**). The RDW is a measure of the size variation of red blood cells, and a high RDW is considered a sign of anemia. In a study comparing the oral microbiome of patients with iron-deficiency anemia (IDA) and healthy controls, IDA was found related to reduced diversity and changes in the relative abundances of several genera (Xi et al., 2019). These results indicate that the oral microbiome of healthy individuals may reflect at least some of the health signatures related to the immune system or anemia.

Among the metabolic biomarkers, fasting glucose explained most of the variation in the microbiome and was found to be related to the abundance of a large number of genera. Associations between type 2 diabetes (T2D) and the oral microbiome have been reported in a number of studies (Long et al., 2017; Chen et al., 2020; Matsha et al., 2020). However, our results indicate that even in non-diabetic patients, the oral microbiome changes with fasting glucose levels. Interestingly, there is a clear two-way relationship between diabetes mellitus and periodontitis (Lalla and Papapanou, 2011), and thus, the dysbiosis of oral microbiome can be associated with both diseases. In this study, increased glucose levels were associated with higher abundances of *Treponema*, *Aggregatibacter*, and *Filifactor*. Among them, *Treponema* is known as a major periodontal pathogenic bacteria and its abundance was higher in periodontitis than in periodontally healthy controls (Patini et al., 2018). In a recent study comparing the oral microbiome of patients with T2D and periodontitis with that of systemically and periodontally healthy controls, *Treponema* was significantly more abundant in patients with T2D than in controls and exhibited positive correlations with blood glucose levels (Vieira Lima et al., 2022). Therefore, *Treponema* may play an important role not only in the development of periodontitis but also in glycemic controls, although the mechanisms of action need to be further investigated.

Moreover, the oral microbiome of the participants in this study could be clustered into two groups. The genera that were differentially abundant in these two clusters were core members of the oral microbiome. These bacteria may have different functions, and thus, the oral microbiomes belonging to the two clusters may differentially influence oral health or systemic health. By clustering the samples according to the oral microbiome composition, we can simplify the oral microbiome type. As RDW was significantly associated with oral microbiome clusters, it would aid identifying people with a higher risk of anemia based on cluster information.

A limitation of this study is that we did not assess the oral health of the participants, although they were apparently healthy and had no significant systemic diseases. Therefore, the oral health of the participants may or may not be healthy. The oral microbes found to be associated with the clinical biomarkers herein may also be associated with oral health. For example, *Dialister* and *Lactobacillus* were found to be associated with TLC and WBC, respectively, in this study, but the associations of these microbes with dental caries has been reported (Wang et al., 2017). The relationships among these blood biomarkers, oral microbes, and oral health needs further investigation. Furthermore, we collected blood samples after at least 8 h of fasting, in order to present the associations of fasting glucose or fasting insulin with the oral microbiome; however, these measures only describe a specific time point. HbA1c indicates an average blood glucose level over the past 2–3 months, while glucose fluctuations measured over 24 h by continuous glucose monitoring describe daily variations in blood glucose levels. These measures would be helpful to assess individuals’ health condition including glucose metabolism in more detail. Thus, further studies are needed to measure not only fasting glucose but also HbA1c and glucose fluctuations over 24 h and to analyze their associations with the oral microbiome.

This study suggests that the oral microbiome may affect specific health conditions or be affected by these factors. A long-term follow-up study will help determine the direction of these relationships. Furthermore, blood biomarkers would be affected by several factors that vary among individuals. The individual variance can be reduced through longitudinal (or time series) monitoring of an individual’s biomarkers, further clarifying microbiome-blood biomarker relationship. In addition, the data generated herein along with future studies investigating clinical association in various diseases could be helpful to develop non-invasive diagnostic models based on the oral microbiome.

## Data availability statement

The datasets presented in this study can be found in online repositories. The names of the repository/repositories and accession number(s) can be found below: https://www.ebi.ac.uk/ena, PRJEB57967.

## Ethics statement

The studies involving human participants were reviewed and approved by the Institutional Review Board of the Chung-Ang University Hospital (2070-005-429). The patients/participants provided their written informed consent to participate in this study.

## Author contributions

MYL and Y-DN conceived of and designed the study. J-HK collected the human saliva samples and clinical metadata. MYL analyzed the data and wrote the manuscript. All authors reviewed and approved the final manuscript.
